# Role of RNA-binding proteins during the late stages of *Flavivirus* replication cycle

**DOI:** 10.1186/s12985-020-01329-7

**Published:** 2020-04-25

**Authors:** Mayra Diosa-Toro, K. Reddisiva Prasanth, Shelton S. Bradrick, Mariano A. Garcia Blanco

**Affiliations:** 1 Programme in Emerging Infectious Diseases, Duke-NUS Medical School, Singapore, Singapore; 2 Department of Biochemistry and Molecular Biology, University of Texas Medical Branch, Galveston, TX USA; 3 Global Health, Surveillance & Diagnostics Group, MRIGlobal, Kansas City, MO USA; 4 Institute for Human Infections and Immunity, University of Texas Medical Branch, Galveston, TX USA

**Keywords:** RNA-binding proteins, *Flavivirus* infection, Viral assembly, RNA export, Exosomes

## Abstract

The genus *Flavivirus* encompasses several worldwide-distributed arthropod-borne viruses including, dengue virus, Japanese encephalitis virus, West Nile virus, yellow fever virus, Zika virus, and tick-borne encephalitis virus. Infection with these viruses manifest with symptoms ranging from febrile illness to life- threatening hypotensive shock and encephalitis. Therefore, flaviviruses pose a great risk to public health. Currently, preventive measures are falling short to control epidemics and there are no antivirals against any *Flavivirus*.

Flaviviruses carry a single stranded positive-sense RNA genome that plays multiple roles in infected cells: it is translated into viral proteins, used as template for genome replication, it is the precursor of the subgenomic flaviviral RNA and it is assembled into new virions. Furthermore, viral RNA genomes are also packaged into extracellular vesicles, e.g. exosomes, which represent an alternate mode of virus dissemination.

Because RNA molecules are at the center of *Flavivirus* replication cycle, viral and host RNA-binding proteins (RBPs) are critical determinants of infection. Numerous studies have revealed the function of RBPs during *Flavivirus* infection, particularly at the level of RNA translation and replication. These proteins, however, are also critical participants at the late stages of the replication cycle. Here we revise the function of host RBPs and the viral proteins capsid, NS2A and NS3, during the packaging of viral RNA and the assembly of new virus particles. Furthermore, we go through the evidence pointing towards the importance of host RBPs in mediating cellular RNA export with the idea that the biogenesis of exosomes harboring *Flavivirus* RNA would follow an analogous pathway.

## Background

### Introduction

The *Flaviviridae* is a family composed of a large number of enveloped positive-strand RNA viruses, many of which pose serious risks to human health on a global scale. This virus family name is derived from the prototype member: the deadly yellow (*flavus*) fever virus (YFV). In 1882, Carlos Finlay suggested that *Culex cubensis* (now known as *Aedes aegypti*) was the mosquito responsible for the transmission of yellow fever, but he was unable to prove his hypothesis [1]. Almost two decades later, in 1901, Walter Reed’s research proved that yellow fever was indeed transmitted by *Aedes aegypti* mosquitoes and caused by a filterable agent found in the blood of infected patients [2]. Decades later the causative virus was isolated and, with the advent of tissue culture methods, passaged extensively by Max Theiler and colleagues, leading to isolation of an attenuated strain (17D) that would later serve as a highly effective vaccine and earn Theiler a Nobel Prize in Physiology or Medicine [3].

The most recent classification of the *Flaviviridae* by the International Committee on Taxonomy of Viruses names 89 species divided between four genera within the family: *Flavivirus*, *Hepacivirus*, *Pegivirus* and *Pestivirus*. This review will focus on the genus *Flavivirus*, which is subdivided into four ecological groups: mosquito-borne, tick-borne, insect-specific and no known arthropod vector flaviviruses [4, 5]. The vast majority of research relevant to this review has been performed with mosquito and tick-borne flaviviruses including dengue virus (DENV), Japanese encephalitis virus (JEV), West Nile virus (WNV), YFV, Zika virus (ZIKV), and tick-borne encephalitis virus (TBEV). These viruses are of clinical importance as they cause major outbreaks with a variety of disease symptoms including hemorrhagic fever and encephalitis [6]. DENV in particular is a significant human health concern as it is ubiquitous in the tropics and estimated to cause nearly 100 million symptomatic infections per year [7]. The severe manifestation of human DENV infection is dengue hemorrhagic fever and dengue shock syndrome, which can be lethal, especially for patients lacking supportive care.

Vector control strategies are the main countermeasures to limit the burden of *Flavivirus*-related diseases; however, traditional methods have failed mainly due to insecticide resistance [8]. Furthermore, although several trials involving mosquito population suppression/replacement are undergoing [9], these are likely to pose complex challenges mainly related to implementation, coverage and public acceptance. Currently, effective vaccines are in use for YFV, JEV and TBEV [10–12]. The development of an effective vaccine against DENV has proven challenging, with recent concerns raised regarding the safety of the only licensed vaccine [13, 14]. Nonetheless, encouraging clinical trials suggest the development of a safe and effective tetravalent DENV vaccine in the near future [15]. At this time, there is no specific antiviral therapy against any virus of the *Flavivirus* genus. Thus, although progress has been made to limit the burden of flaviviruses epidemics*,* more work needs to be done. In particular, there are concerns about emerging viruses with novel pathogenic potential, exemplified by the recent ZIKV pandemic and its association with birth defects [16].

### Characteristics of Flavivirus virions and genome organization

Flaviviruses are enveloped viruses in which the viral RNA (vRNA) and capsid (C) protein are surrounded by a lipid bilayer derived from the host cell. Embedded in this outer layer, two viral glycoproteins are found: envelope (E) and Membrane (M) [17]. The structures of multiple flaviviruses have been solved and the arrangement and stoichiometry of M and E have been well characterized in both mature and immature virions; the latter have an uncleaved version of M referred to as prM [18, 19]. Within the interior of the virion is the nucleocapsid, formed by the positive-sense single stranded vRNA genome associated with C protein. Infectious *Flavivirus* particles are relatively uniform in size (~ 50 nm in diameter) and density (1.19 to 1.23 g/cm^3^) [17], but infected cells also produce smaller (~ 30 nm in diameter), non-infectious enveloped particles that contain M and E proteins but lack nucleocapsid [20].

The *Flavivirus* genome is around 11 kb in length, carries a type 1 cap (m7GpppAmp) structure at the 5′ end and lacks a poly(A) tail at the 3′ end [21, 22]. The single open reading frame (ORF) is flanked by highly structured 5′ and 3′ untranslated regions (UTRs) involved in translation, replication and likely packaging of the vRNA. The 5′ UTR is relatively short (~ 100 nt) and carries a large stem-loop (SLA) that functions as promoter for the viral polymerase, NS5, to initiate RNA synthesis at the 3′ end of a ‘circularized’ genome [23]. The 3′ UTR is larger (~ 400 to 700 nt) and includes three distinct domains. Domain 1, which is the least conserved among flaviviruses, is known as the ‘variable region’ and contains two stem-loop structures (SLI and SLII) that form pseudoknots with adjacent sequences; domain 2 includes either one (e.g., ZIKV and YFV) or two (e.g., DENV and JEV) conserved dumbbell structures (DB1 and DB2) [24]. The structures present in domain 1 facilitate but are considered dispensable for replication [25–27]. Finally, domain 3, the most conserved region of the 3′ UTR, contains a complementary sequence element (CS1) followed by a terminal stem-loop structure (3′ SL) [22, 28]. Both, CS1 and portions of the 3′ SL are complementary to sequences present at the 5′ end and thus, allow the circularization of the RNA genome, a step required prior to vRNA replication [23].

In addition to its role during vRNA replication, the *Flavivirus* 3′ UTR is the precursor of the subgenomic flaviviral RNA (sfRNA). sfRNA results from partial degradation of vRNA by the host 5′ ➔ 3′ exoribonuclease XRN1, which stalls at exonuclease resistant RNA structures (xrRNAs) present in the 3′ UTR [29, 30]. The *Flavivirus* 3′ UTR contains up to four xrRNAs, corresponding with compactly folded RNA elements found in the SL and DB structures. In some instances, XRN1 slips through the first xrRNA and stalls at a downstream structure giving rise to sfRNAs of different lengths [31, 32]. The production of sfRNA is independent of virus replication and viral proteins and is conserved in all flaviviruses studied thus far [29, 33, 34]. Its functions relate to pathogenicity, inhibition of antiviral responses, increased viral transmission and higher replicative fitness [29, 35–38].

### Flavivirus replication cycle

Flaviviruses enter a susceptible host cell by receptor-mediated endocytosis. There is not a single receptor for these viruses; rather binding of E to various membrane-bound molecules has been shown to mediate entry. Possible receptors include α_v_β_3_ integrins, C-type lectin receptors (CD206, CD209, CLEC5A), TIM/TAM phosphatidylserine receptors, heat shock proteins 70 and 90, among others [39–41]. In addition, virus particles opsonized with immunoglobulins, which are present during secondary DENV infections, mediate entry in cells bearing Fc receptors [42]. Internalization of the viral particle occurs mainly through clathrin-mediated endocytosis which delivers virus particles to the early endosome [43, 44]. Maturation from early to late endosomes with a concomitant drop in pH favours conformational changes in the viral E protein resulting in its fusion with host cell membranes [45]. Following the fusion step, the viral RNA (vRNA) is released into the cytoplasm. The process of uncoating, e.g. the disassembly of the viral ribonucleoprotein (RNP) complex, is not completely understood, but it has been suggested that a non-degradative ubiquitination of C, mediated by E1-activating enzyme is required for genome uncoating [46]. We have previously proposed a model in which elongating ribosomes drive the release of C from the vRNA [47], but this remains to be investigated.

Once in the cytoplasm the vRNA is transported to the endoplasmic reticulum (ER) where translation takes place [48]. The vRNA has a type 1 cap structure and therefore, translation is considered to be cap-dependent, like the majority of the eukaryotic mRNAs [49]; however, non-canonical mechanisms of translation initiation have also been described for DENV [50–52]. Translation and concomitant processing of the ER-bound polyprotein generates three structural proteins (C, prM/M and E) and seven non-structural (NS) proteins [53]. The NS proteins include NS3 and NS5, which carry enzymatic activities (NS3 is a protease and helicase while NS5 is a RNA-dependent RNA polymerase (RdRp) and methyltransferase), and NS1, NS2A, NS2B, NS4A, and NS4B, which participate in vRNA replication and virion assembly [54].

Viral non-structural proteins mediate vRNA replication, which occurs within virus-induced ER invaginations, known as vesicle packets (VPs). The components of the replication complex, vRNA, NS proteins and possibly host factors, are found inside VPs [55]. VPs are connected to the cytoplasm via a pore, through which other factors needed for replication such as nucleotides can enter [55, 56]. Using the viral genome as template, NS5 synthesizes antisense (−)RNA, generating double stranded (ds) RNA replicative intermediates. The (−)RNA serves as template for additional positive-sense vRNA synthesis.

**Fig. 1:**
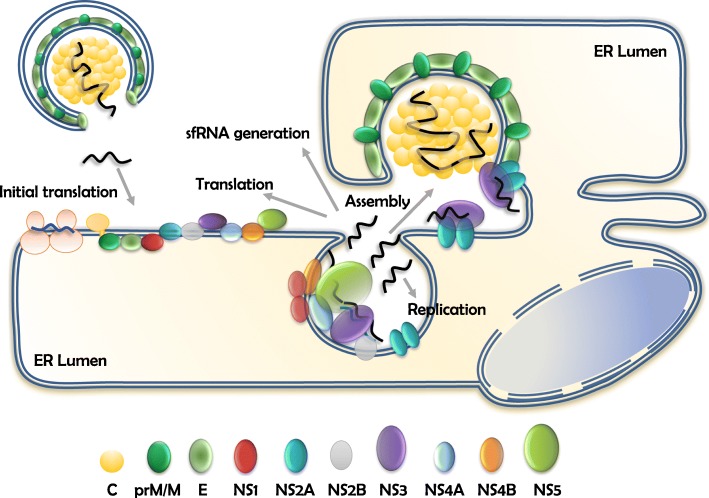
RNA transactions in *Flavivirus*-infected cells. Flaviviruses carry a positive-sense RNA that is immediately translated upon delivery into a susceptible host cell. Translation takes place at the ER membrane and generates a single polyprotein processed into three structural proteins (C, prM/M and E) and seven non-structural (NS) proteins. Replication of vRNA takes place inside virus-induced invaginations known as vesicle packets (VPs). Newly synthesized vRNA function as template for translation, further replication, generation of sfRNA molecules or is assembled into new virus particles. Assembly takes place at locations juxtaposed to the VPs. The viral proteins NS2A and NS3 have been implicated in the assembly of new virus particles

Newly synthesized vRNAs exit through the pore of VPs and serve multiple purposes. They are used to translate viral proteins, function as template for (−)RNA synthesis to generate additional genomes, become the precursors of sfRNA molecules, or are selectively packaged into viral particles [57, 58]. All these processes are tightly coordinated in time and space but little is known about the mechanisms that determine the fate of vRNA molecules at this point. vRNA molecules destined to be part of virions are relocated to the assembly sites (Fig. 1), where the nucleocapsid is formed and newly assembled virus particles containing E and prM bud into the ER lumen (details of this step will be discussed below). These immature virions are transported via the secretory pathway where the slightly acidic pH (~ 5.8–6.0) of the *trans*-Golgi network triggers conformational changes of E/prM [18, 19]. Such changes expose a cleavage site for the cellular protease furin that releases the pr-peptide from the M protein [59]. The cleaved pr fragment remains associated with the virion until the virus is secreted to the extracellular environment [60].

## Main text

### Role of viral and cellular RBPs during the late stages of the replication cycle

#### Viral RNA encapsidation

The molecular details leading to *Flavivirus* vRNA encapsidation and assembly into new virions remains largely unknown. Notably, the process is thought to be highlys specific, as only a single full length (+)vRNA molecule is packaged into virions [61], whereas sfRNA molecules, (−)RNA and host RNAs are excluded. The selective packaging of single stranded RNA viruses is often attributed to the presence of packaging signals, specific sequences and/or secondary structures that promote interaction with C proteins [62]. The C-binding signals have not been identified for flaviviruses. Therefore, non-specific electrostatic interactions between highly basic C protein and negatively charged vRNA are likely to mediate *Flavivirus* nucleocapsid formation [63]. Notably, in spite of C being the least conserved of the *Flavivirus* viral proteins (less than 40% sequence identity), the charge distribution is similar among the viruses in the genus [64]. Indeed, mutational analysis of two conserved clusters of basic amino acids revealed that no specific residues were required for DENV virus production as long as the number of positive charges were preserved [65]. The study of *Flavivirus* particles by cryo-electron microscopy did not visualize an organized nucleocapsid suggesting that it does not have the same symmetry as the envelope or that it is stochastically arrayed, which could be mediated by non-specific interactions between C and vRNA [66, 67].

Nucleocapsids have not been visualized in *Flavivirus*-infected cells suggesting that encapsidation is rapid and/or concurrent with replication and budding. Indeed, Khromykh and collaborators showed that packaging of Kunjin (KUN) WNV vRNA is coupled to active vRNA replication [68]. For their study, the authors designed a DNA-based KUN replicon that allows the continuous transcription by cellular RNA polymerase II and accumulation of replication-competent (wild-type) and replication-deficient (RdRp-defective) KUN RNAs. As expected, transfection of wild-type DNA constructs yielded replicating KUN RNA and secretion of infectious virions. On the other hand, transfection of the RdRp-defective construct resulted in the cytoplasmic accumulation of RdRp-defective RNA and the secretion of structural proteins; however, this RNA was not packaged into secreted virus particles. Notably, when the RdRp-defective plasmid was transfected into helper BHK-21 cells, which persistently express KUN replicon RNA and thus provide wild-type replicative proteins, the RdRp-defective RNA was packaged and secreted into virus particles [68]. Their results strongly suggest that replication and assembly are coupled. Coupling of vRNA replication with assembly plausibly gives specificity to the encapsidation process and its straightforward to envision a model wherein the vRNA will exit through the pore of the VP which is juxtaposed to the assembly sites at the ER [55].

Further specificity to the encapsidation process could be given by RNA modifications or by interactions of C and vRNA with viral or host proteins. For example, the vRNA of DENV, ZIKV and Hepatitis C virus (HCV), a member of the *Hepacivirus* genus of the *Flaviviridae* family, are targets for N6-methyltransferases within the E and RdRp regions [69]. The presence of this modification in the E region was shown to abrogate HCV vRNA packaging without altering replication, suggesting that the formation of the HCV nucleocapsid is regulated in such way that vRNA lacking the N6-methylation is preferentially packaged into budding virions [69]. Nevertheless, it remains to be established whether this modification is implicated in the regulation of DENV and ZIKV vRNA packaging.

Lastly, several host factors have been found to interact with *Flavivirus* C protein with the potential of modulating the encapsidation process. For example, Li and collaborators identified 70 WNV C-interacting partners, the majority of which are involved in RNA processing [70]. In the same study it is shown that despite the limited sequence similarity between the C protein from DENV, WNV and ZIKV, there is a significant overlap between their interacting host factors [70]. One of these is the exon-junction complex recycling factor PYM1, a protein involved in non-sense mediated decay (NMD). The authors showed that the NMD pathway is antiviral against WNV, DENV and ZIKV, and that WNV antagonizes this response by binding of C to PYM1, which protects vRNA from NMD-mediated degradation [70]. Other RBPs such as Caprin1, nucleophosmin and hnRNPK were previously shown to bind JEV and DENV C protein, respectively [71–74]. These host factors are important for infection given that their knockdown reduced virus titers; however, whether the encapsidation and/or assembly steps were affected by these proteins was not determined. On the contrary, DDX56 and nucleolin are host RBPs described to bind C protein and specifically affect the assembly step of WNV and DENV, respectively [75–78]. We refer to these proteins in a later section.

#### Post-replicative functions of NS proteins

The function of *Flavivirus* NS proteins is best characterized during vRNA replication, however, NS proteins are also required for the assembly and egress of virus particles [79]. Studies on the molecular mechanisms by which NS proteins regulate the late stages of the replication cycle are limited because genetic perturbations of most NS proteins lead to the abrogation of RNA replication. Here, we will discuss recent data pointing towards the essential functions of NS2A and NS3 for assembly.

##### NS2A

*Flavivirus* NS2A is a small (~ 22 KDa), hydrophobic protein generated after processing of the viral polyprotein by an unknown host protease and the viral protease at the NS1-NS2 and NS2A-NS2B boundaries, respectively [80]. It contains eight segments which have been named predicted transmembrane segments (pTMS), although only five of them truly transverse the ER membrane. The N-terminus, pTMS1 and pTMS5 localize to the ER lumen and do not interact with its membrane; pTMS2 is in the ER lumen and interacts peripherally with the ER membrane; pTMS3, 4, 6, 7 and 8 are integral transmembrane domains and a short C-terminus localizes to the cytoplasm [81]. Several reports have mapped NS2A residues that are determinants for viral assembly and egress. It has been shown that the mutation Lys-90-Ser or the triple mutation Arg-22-Ala/Lys-23-Ala/ Arg-24-Ala in YFV NS2A does not affect the accumulation of viral protein or vRNA, but decreases the production of infectious virions [82, 83]. Interestingly, the Lys-90-Ser mutation had no effect on the production of VLPs that lack capsid and vRNA, suggesting that NS2A participates in the incorporation of vRNA into the budding particle [82]. Similarly, an Ile-59-Asn mutation impaired the assembly of KUN WNV. The mutation was associated with the absence of the ER membrane remodelling present in cells transfected with a wild-type infectious RNA replicon. Importantly, a compensatory mutation Thr-149-Pro restored the membrane remodelling and virus production to levels similar to wild-type [84, 85].

More recently, a detailed mechanism of the function for NS2A during DENV virus assembly was described by Xie and collaborators [86, 87]. The authors reported a set of mutations that specifically impaired virus assembly without affecting vRNA synthesis. These mutants, which were incompetent for viral assembly, (Gly-11-Ala, Glu-20-Ala, Glu-100-Ala, Gln-187-Ala, and Lys-188-Ala), could be rescued in *trans* by expressing wild-type NS2A or a mutant unable to support vRNA replication [86]. Further studies demonstrated that despite similar levels of viral protein synthesis and ER membrane rearrangements between wild-type RNA and the Gly-11-Ala mutant, the latter failed to form viral particles, as assessed by transmission electron microscopy, and reduced release of extracellular vRNA [87]. Moreover, the Gly-11-Ala mutant showed a significant decrease in the C-prM cleavage which resulted in an altered cellular localization of C, a decreased expression of E and an abrogation of prM expression. This defect in C-prM cleavage was explained by enhanced binding of mutant NS2A to prM on the ER lumen, which pulls the C anchor towards the ER membrane making it inaccessible to NS2B/NS3 in the cytoplasmic side. This defect was rescued by *trans* complementation with wild-type NS2A and several mutations in C which shifted the C-prM cleavage site by two amino acids. Similarly, ZIKV NS2A was shown to independently bind prM/E and NS2B/NS3 [88] which would facilitate the recruitment of these complexes to the virion assembly site by oligomerization of NS2A.

Several studies have suggested the involvement of NS2A in the transport of vRNA from the replication complexes to the sites of viral assembly, whereby NS2A functions as a viroporin [85, 89]. Viroporins are hydrophobic proteins that oligomerize at cellular membranes resulting in the formation of an hydrophilic channel that alters membrane permeability, vesicular trafficking and Ca^+ 2^ homeostasis [90]. Indeed, overexpression of DENV and JEV NS2A in *E. coli* resulted in increased membrane permeability and lysis [89, 91]; however, more recent studies point towards a different mechanism by which NS2A transports newly synthesized vRNA. In their study, Xie and collaborators [87] showed that NS2A specifically binds to DENV vRNA, with a cytoplasmic loop of NS2A (amino acids 93 to 100) interacting with the last 285 nucleotides of DENV 3′ UTR. Similar findings were reported by the same group for ZIKA NS2A, where the cytoplasmic loop (residues 97 to 104) was shown to interact with the last 333 nucleotides from the 3′ UTR [88]. As no RNA packaging signal has been identified for *Flavivirus* assembly, the authors hypothesize that complex structures located in the 3′ UTR of DENV and ZIKA serve as signals for NS2A to recruit newly synthesized vRNA from the replication complex to the viral assembly site. Because mutations that alter virus assembly do not influence the interaction with DENV or ZIKA 3′ UTR, the authors propose a model in which different molecules of NS2A recruit C-prM-E, NS2B/NS3 and vRNA to the virion assembly site. These molecules are brought together due to oligomerization of NS2A after which processing of the structural proteins occurs yielding free C that binds to vRNA to form the nucleocapsid on the cytoplasmic side. The nucleocapsid is then enveloped by E and prM leading to virion formation and budding into the ER lumen [87, 88]. Although the proposed model is very compelling, it remains to be addressed whether interaction of the vRNA with C will lead to its detachment from NS2A, or whether NS2A is incorporated into the virion. Likewise, *Flavivirus* infection produce large amounts of sfRNA, as these molecules derive from the 3′ UTR from the vRNA, an important question is whether NS2A can bind sfRNA molecules free in the cytoplasm and whether or not these are incorporated into virions.

##### NS3

*Flavivirus* NS3 (~ 70 KDa) is a multifunctional protein with two domains, the N-terminal domain has chymotrypsin-like serine protease activity and the C-terminal domain has helicase, ATPase and RNA 5′ triphosphatase activities [92]. In addition, an ATP-independent RNA annealing activity has also been reported [93]. The protease activity of NS3 is required in *cis* and *trans* for processing of the viral polyprotein, whereby NS2B functions as cofactor. The precise function of the helicase domain during vRNA replication is not known, but it is presumed that it resolves secondary structures present in the vRNA, displaces other RNA-binding proteins to remodel the viral RNP, and/or separates the dsRNA intermediate formed upon replication [94]. Together NS3 and NS5 have all the enzymatic activities required for the replication of *Flavivirus* vRNA, and recently a hand full of studies suggest that the function of NS3 extends outside of the replication complex.

Efficient *Flavivirus* assembly requires an obligatory sequential cleavage at the C-prM junction by NS2B-NS3 protease at the C cytosolic side and then by cellular signalase at the ER lumen side. The coordinated processing releases the mature C protein required for encapsidation [95, 96]. In addition to enabling the processing of C, NS3 appears to have other roles during viral assembly. As Pijlman and collaborators demonstrated, KUN WNV NS3 mutants with defects for C processing and RNA replication can be rescued in *trans*, while mutations affecting packaging cannot be complemented with functional NS3 [97]. The authors proposed a model in which newly synthesized NS3 associates with the progeny vRNA template at the same time that it interacts with other viral (or host) proteins. This viral RNP complex is responsible for the re-localization of vRNA to the sites of encapsidation/assembly [97]. Though proposed more than a decade ago, this hypothesis remains valid particularly in light of the new function discovered for NS2A described in the previous section. NS3 is also required for the packaging of YFV vRNA because a single mutation (Trp-349-Ala) in the helicase domain resulted in less infectious virus release without affecting the expression of viral proteins, vRNA replication and release of subviral particles lacking C protein and vRNA [98]. Interestingly, *trans* complementation with the NS3 mutants S138A or R461Q in which the protease and the helicase are inactive, respectively, rescued virus production, suggesting that the function of NS3 during virus assembly is independent from its known enzymatic activity. Lastly, structural studies suggest that binding of NS3 to specific 5′ UTR sequences could function as a molecular signature to guide newly synthesized vRNA out of the replication complex [99]. Nevertheless, biophysical and biochemical evidence to support this hypothesis is lacking.

Interactions of NS3 with several host factors have also been reported [70, 100]. Of importance for assembly, YFV and DENV NS3 were found to interact with apoptosis linked gene-2-interacting protein X (ALIX) [101, 102]. In both cases knock out of ALIX or transfection with truncated versions of the protein resulted in the inhibition of virus release without affecting vRNA replication. ALIX is an accessory protein for the endosomal sorting complex required for transport (ESCRT) machinery that mediates membrane deformation favouring budding away from the cytoplasm [103]. Several ESCRT subunits are required for the membrane deformation during budding of the *Flavivirus* viral particle but not for the formation of VPs [104]. Because ALIX plays a role in concentrating cargoes that are incorporated into budding vesicles, is tempting to speculate that its interaction with NS3 mediates proper assembly of viral particles, however, this remains to be examined.

#### Host cell RBPs with potential roles in virus assembly

Numerous studies have been undertaken in order to characterize the mechanism by which cellular RBPs regulate *Flavivirus* replication cycle and we refer the reader to recent reviews on this topic [57, 58, 105]. In most cases, cellular RBPs have been shown to play roles in vRNA translation and replication; however, few of these proteins have been shown to affect *Flavivirus* infection beyond the replication step. One such example is DDX56, a nucleolar RNA helicase member of the DEAD box protein family [106]. Hobman and collaborators have comprehensively studied the mechanism by which DDX56 regulates the packaging of WNV vRNA. DDX56 was initially identified as a C-interacting partner by the means of yeast two-hybrid screening followed by immunoprecipitation. Importantly this interaction was shown to be RNA-independent [75]. Knockdown of DDX56 in several human-derived cell lines resulted in a significant decrease of infectious WNV in the supernatant. The reduced infectivity was associated with a decrease in the amount of vRNA being packaged and secreted in virions given that same levels of WNV C protein were secreted from control and DDX56-knockdown cells [75]. The authors then showed that infectious virus production is rescued in *trans* by wild-type DDX56 but not by the mutants D166N and E167Q in which the helicase activity is abrogated. Furthermore, over-expression of the C-terminus of DDX56, which was identified as the C-interacting domain, reduced the amount of secreted vRNA but not secreted C. Therefore, the authors proposed that interaction of DDX56 with C enables loading of vRNA during the assembly of the virus particle [76]. Given these results and because during WNV infection DDX56 relocates from the nucleus to the cytoplasm [75], it was presumed that interaction between WNV C and DDX56 would occur in both cellular compartments. However, recent data indicate that such interaction only occurs in the nucleus [77]. In contrast, a large proportion of cytoplasmic DDX56 colocalized with WNV E protein at the ER, suggesting that DDX56 indeed localizes to virus assembly sites. Nevertheless, how the C-DDX56 and E-DDX56 nuclear and cytoplasmic interactions, affect the packaging of vRNA into virions remains unknown.

Nucleolin, a highly conserved nuclear phosphoprotein, is another RBP shown to mediate *Flavivirus* assembly [78]. Balinsky et al found that nucleolin interacts with DENV C in both nucleus and cytoplasm in an RNA-independent manner. The interaction, which was verified in DENV-infected cells as well as in cell-free reactions, was abolished by the use of a nucleolin-binding aptamer (AS1411). Furthermore, siRNA-mediated knockdown of nucleolin as well as treatment with AS1411 resulted in a significant decrease of DENV titers despite similar levels of intracellular vRNA and viral proteins and secreted E and C. These results resemble those of DDX56, however, chemical interference of nucleolin slightly reduced the number of vRNA copies in supernatant and nucleolin knockdown did not affect secreted vRNA at all. The authors failed to investigate why siRNA-mediated knockdown of nucleolin resulted in lower virus titers without altering supernatant vRNA. It would be interesting to test whether the lost interactions between nucleolin and C increases the ratio of immature viruses, thus generating virus progeny with the same vRNA levels but with different infectivity.

Recently, our group uncovered two other RBPs that are required beyond the replication step of *Flavivirus* vRNA. One of them is Y-box-binding protein 1 (YBX1), a highly conserved cold shock domain protein that binds to DENV vRNA [38, 107, 108]. We previously reported that siRNA-mediated knockdown of YBX1 increased the intracellular DENV vRNA levels while the release of infectious particles was impaired, suggesting a role for YBX1 at the stages of assembly/secretion [108]. We have now expanded these findings and confirmed that CRISPR-Cas9-mediated knockout of YBX1 results in a significant decrease of infectious titers, vRNA and structural proteins in supernatants, whereas no changes in intracellular viral proteins are observed (Diosa-Toro et al, unpublished results). Currently, we hypothesize that assembly of infectious particles is impaired in cells lacking YBX1. The second protein complex affecting late steps is Topoisomerase 3B (Top3B)-Tudor Domain Containing protein 3 (TDRD3) complex. The Top3B-TDRD3 is a dual activity complex that is essential for promoting both transcription and translation [109, 110]. CRISPR/Cas9 based deletion of Top3B-TDRD3 complex components did not affect *Flavivirus* translation and replication, whereas it impaired the production of infectious virus particles. We further characterized that depletion of Top3B-TDRD3 complex did not inhibit particle release (Prasanth et al, unpublished results).

### Role of RBPs in determining the extracellular fate of RNA molecules

**Fig. 2:**
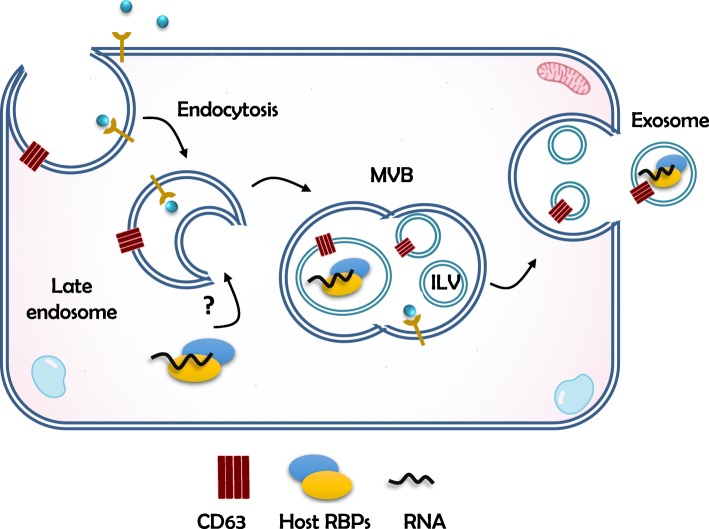
Exosome biogenesis. Late endosomes sequester cargo molecules in intraluminal vesicles (ILVs) generating multivesicular bodies (MVBs). MVBs fuse with lysosomes for degradation or fuse with the plasma membrane releasing exosomes to the extracellular milieu. The intersection between cytoplasmic ribonucleoprotein complexes and endosomes might be a step prior the export of RNA molecules via exosomes

Progeny virus particles are considered the sole mechanism for the dissemination of flaviviruses. Nevertheless, cell-derived extracellular vesicles carrying *Flavivirus* vRNA have recently been shown to be infectious [111–116], thus representing an alternate mode of virus dissemination. The pathway leading to the secretion of vRNA independently of virus particles is unknown, but parallels can be drawn with the export of cellular RNAs. In recent years, the literature has seen an explosion of studies on the extracellular RNA species that are found in supernatants from cultured cells and in extracellular compartments in vivo [117–119]. RNA is found outside cells as cargo of extracellular vesicles (EVs) or in tight association with RBPs and/or lipids [117]. EVs are heterogeneous in size, and include apoptotic bodies, microvesicles and exosomes. Microvesicles (100–1000 nm) bud from the plasma membrane, and exosomes (40–100 nm) originate from the endocytic pathway [120]. In the latter, vesicles progress from early to late endosomes, where cargo is sequestered into intraluminal vesicles (ILVs) (Fig. 2). ILVs arise from the inward budding of the outer endosomal membrane, thus, forming a multivesicular body (MVB). MVBs either fuse with lysosomes for degradation or fuse with the plasma membrane in which case, the ILVs are released into the extracellular milieu as exosomes [121]. By transferring a wide range of molecules, such as proteins, RNAs and lipids, exosomes modulate intercellular communication during homeostatic and pathogenic conditions. For example, EVs from infected cells have been shown to harbour antiviral proteins such as APOBEC3G and STING [122, 123], indicating how immune signals can be transferred to elicit responses in neighbouring cells. On the other hand, EVs are also hijacked by viruses to facilitate dissemination.

Studies on extracellular RNAs have primarily focused on microRNAs (miRNAs), small (20–22 nucleotides) non-coding RNAs that target mRNAs and usually induce silencing of gene expression [124, 125]. Exosome-associated miRNAs regulate multiple cellular processes within the recipient cells and are considered potential biomarkers for cancer and metabolic diseases [126–128]. Nevertheless, we lack understanding of the mechanisms that govern sorting of these RNA molecules into exosomes. Because exosomes are enriched in particular classes of RNAs and their content does not always reflect the intracellular RNA pool [129–132], a mechanism by which certain RNAs are selected as cargo for exosomes must be in place. Whether *cis*-acting regulatory sequences/structures on the RNA molecule or *trans*-acting factors – or both- are responsible for the export of RNAs remains largely unknown. Potential *cis*-acting factors are the short motifs GGAG and GGCU that were found enriched in exosomal miRNAs and were specifically recognized by heterogeneous nuclear ribonucleoprotein A2B1 (hnRNPA2B1) in human primary T-lymphocytes [133] and by hnRNP Q in hepatocytes [134]. Nevertheless, not all exosomal miRNAs contain those (or other) motifs. For example, despite lacking particular signals associated with exosomes, miR-223 was found to be specifically sorted into exosomes by YBX1 in HEK293T cells and in cell-free reactions [135]. Further evidence points towards a more general role of YBX1 in the sorting of other highly abundant types of small RNAs such as tRNAs, Y RNAs and Vault RNAs [132]. Because RBPs such as hnRNPA2B1, hnRNP Q and YBX1 do not contain transmembrane domains it remains unclear how they mediate interaction with MBVs. Interestingly, all these proteins localize to cytoplasmic RNP complexes [136–138] and it has been suggested that intersection between RNPs and MBVs is a required step prior to the secretion of RNA molecules [139]. Hence, determinants for secretion are likely cell specific and dependent on factors such as the binding affinity of certain RBPs for particular RNA molecules. Certainly, a wide range of RBPs are also found in exosomes [140], favouring a hypothesis in which RNP complexes carry the signals for secretion.

Besides carrying endogenous molecules, EVs also carry virus particles and infectious genomes. This phenomenon was initially discovered for non-enveloped viruses in which several viral genomes are packaged and released inside EVs, thus mediating *en bloc* viral transmission [141, 142]. Interestingly, several reports indicate that enveloped viruses that are traditionally seen to disseminate as free viral particles, also exploit EVs for their propagation [142] [143]. In particular, cells infected with HCV [144–146], DENV [111–114], ZIKV [115] and TBEV [116] release exosomes that are infectious to recipient cells. These infectious exosomes carry full-length vRNA but do not always contain the structural viral proteins associated with virions, thus suggesting that in *Flavivirus*-infected cells, vRNA molecules are sorted into exosomes independently of the assembly of fully mature particles. It is not known how vRNAs are sequestered into endosomal ILVs, but it is likely that, as in the case of cellular RNAs, specific RBPs would mediate the interaction with MBVs. For example, exosomes from sera of HCV-infected patients were found to contain vRNA, miR-122, HSP90 and the RBP Ago2 [145]. The binding of miR-122 to HCV vRNA in association with Ago2 favors vRNA replication [147, 148] and it was previously shown that HSP90 functions as a chaperone for Argonaute proteins facilitating the loading of small RNAs [149]. It is interesting that these components of cytoplasmic RNPs are present in infectious HCV exosomes. Similarly, it is intriguing that YBX1 and hnRNPA2B1, previously described to mediate the packaging of cellular RNAs into exosomes, have been shown to bind DENV vRNA [107, 108, 150]. Furthermore, DENV, WNV and TBEV vRNA colocalizes with several RBPs present in cytoplasmic RNA granules [35, 151–154], which could potentially facilitate its sorting into exosomes. Collectively, these data highlight a previously overlooked function of cellular RBPs during *Flavivirus* infection. We propose that these proteins do not only favor the assembly and release of traditional virions, but are likely involved in the secretion of alternative infectious units, with potentially important consequences for viral dissemination and immune recognition.

## Conclusions

**Fig. 3:**
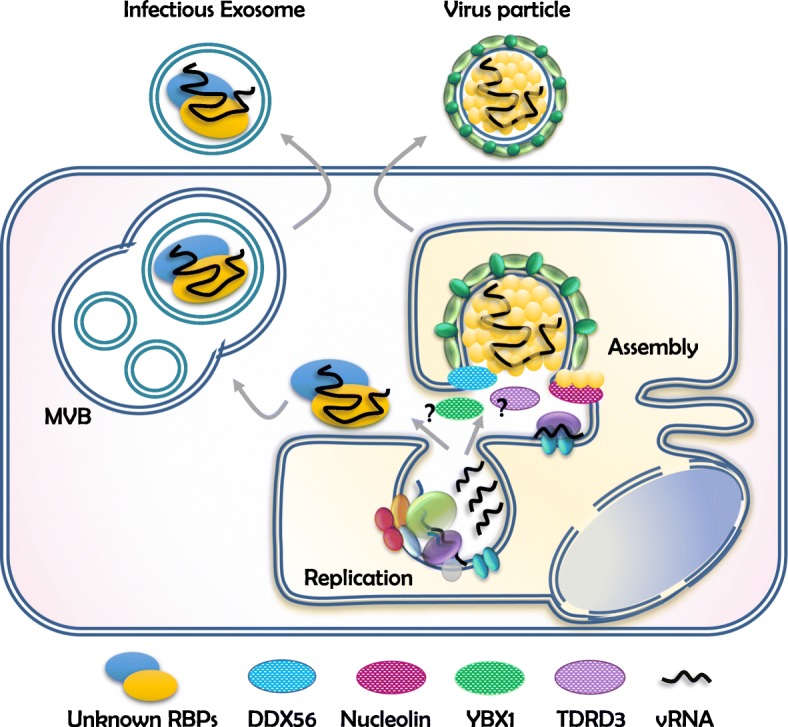
Cellular RBPs participate in the assembly of *Flavivirus* particles and possibly the loading of vRNA into exosomes. In addition to viral-encoded proteins (Fig. 2), host RBPs including DDX56 and nucleolin have been reported to enable *Flavivirus* assembly. Furthermore, our group has discovered that YBX1 and TDRD3 are also required during this step (unpublished results). We propose that RBPs also mediate the sorting of vRNA into exosomes and therefore contribute to an alternative route of virus dissemination

The remarkable multifunctionally of the viral-encoded RBPs and the large diversity of the host RBPs co-opted by flaviviruses highlights the importance of these proteins in determining virus-cell host interactions. Here, we emphasize that in addition to the better characterized functions during vRNA translation and replication, RBPs participate at later stages of the *Flavivirus* replication cycle, particularly, during the packaging of vRNA and assembly into virus particles. Furthermore, we propose that the biogenesis of infectious exosomes, which represent an alternate mode of virus dissemination, relies on the recognition of vRNA by host RBPs (Fig. 3).

In *Flavivirus*-infected cells, newly synthesized RNA is translated to generate new viral proteins, as template to generate additional vRNA molecules, as precursor of sfRNA molecules and is encapsidated into new virus particles. How does the virus coordinates these processes remains largely unknown. The process of assembly is considered highly specific because only positive-sense vRNA molecules are incorporated into budding virions. Remarkably, this is a common assumption that requires experimental verification. Whereas the coupling of replication and assembly confers specificity to vRNA (thus, excluding cellular RNA molecules from being incorporated into virus particles), how negative-sense RNA and sfRNA molecules are excluded from the assembly process remains to be determined. We have to consider, however, that the exclusion of sfRNA from virions, which was reported by our group for DENV2-NGC [35], may not be conserved for all flaviviruses. It is likely that dynamic secondary and tertiary structures adopted by the vRNA at different stages of the replication cycle will affect its interaction with viral proteins and cellular RBPs, thus conferring molecular specificity for different processes. Interestingly, we also lack evidence to confirm another widespread assumption about the protein composition of flaviviruses particles, that is, that only C, prM/M and E are present. Given the critical role of NS2A and NS3 during virus assembly that we summarize in this review, it remains a possibility that these viral RBPs are incorporated into virions. Indeed, the interior structure and composition of *Flavivirus* particles remains elusive and, to the best of our knowledge, proteomic analysis of virus preparations are lacking. For the same reason, host proteins have not been associated with *Flavivirus* virions unlike the related HCV particles, which incorporate host apolipoproteins [155, 156].

Remarkably, host RBPs potentially contribute to the dissemination of *Flavivirus* infection via the incorporation of vRNA into exosomes. How these infectious exosomes are formed and what they contribute to viral dissemination is not known. Taking into account that exosomes may be poorly recognized by the host immune system [157] and that *en bloc* transmission of multiple genomes favors higher replicative fitness [141, 142], a deeper study of the role of exosomes during *Flavivirus* infection and their biogenesis is warranted. It is interesting that viral assembly/release and exosome biogenesis share common features, such as membrane remodelling, loading of specific cargo and fusion with the plasma membrane. Furthermore, host factors from the vesicular trafficking machinery are required for the egress of flaviviruses. For example, the assembly, but not replication, of DENV, JEV and YFV has been shown to require components of ESCRT, which is central to exosome biogenesis [102, 104]. In addition, DENV, WNV and ZIKV have been shown to induce autophagy [158–162], a pathway that was recently described to mediate the loading of RBPs into EVs [163]. Further studies are required to establish whether flaviviruses evolved to co-opt exosomes as an alternative route of dissemination, but it is clear that RBPs guide the fate of vRNA molecules and thus, potentially determine the mechanism by which viral infection is disseminated.

## Data Availability

Not applicable.

## Abbreviations

ALIX: Apoptosis linked gene-2-interacting protein X
C: Capsid
CD: Cluster of differentiation
CLEC5A: C-type lectin domain family 5 member A
CS: Complementary sequence
DB: Dumbbell
DDX56: DEAD-box helicase 56
DENV: Dengue virus
dsRNA: Double stranded RNA
E: Envelope
ER: Endoplasmic reticulum
ESCRT: Endosomal sorting complex required for transport
EVs: Extracellular vesicles
HCV: Hepatitis C virus
hnRNP: Heterogeneous nuclear ribonucleoprotein
ILV: Intraluminal vesicle
JEV: Japanese encephalitis virus
KUN: Kunjin
M: Membrane
miRNAs: micro RNA
MVB: Multivesicular body
NMD: Non-sense mediated decay
NS: Non-structural protein
ORF: Open reading frame
pTMS: predicted transmembrane segments
RdRp: RNA-dependent RNA polymerase
RNP: Ribonucleoprotein complex
sfRNA: subgenomic flaviviral RNA
SL: Stem-loop
TAM: Tyro3, Axl, and Mer
TBEV: Tick-borne encephalitis virus
TDRD3: Tudor Domain Containing 3
TIM/TAM: T-cell immunoglobulin and mucin domain 1
TMs: Transmembrane domains
VP: Vesicle packets
vRNA: viral RNA
WNV: West Nile virus
xrRNA: exonuclease resistant RNA structure
YBX1: Y-box-binding protein 1
YFV: Yellow fever virus
ZIKV: Zika virus
